# Vpx rescue of HIV-1 from the antiviral state in mature dendritic cells is independent of the intracellular deoxynucleotide concentration

**DOI:** 10.1186/1742-4690-11-12

**Published:** 2014-02-01

**Authors:** Christian Reinhard, Dario Bottinelli, Baek Kim, Jeremy Luban

**Affiliations:** 1 Department of Microbiology and Molecular Medicine, University of Geneva, 1 Rue Michel Servet, Geneva 4 CH-1211, Switzerland; 2 School of Medicine and Dentistry, University of Rochester 601, Elmwood Ave Box 672, Rochester, NY 14642, USA; 3 Program in Molecular Medicine, University of Massachusetts Medical School, 373 Plantation Street, Biotech II, Suite 319, Worcester, MA 01605, USA

**Keywords:** HIV-1, Vpx, SIV, Dendritic cell, SAMHD1, Interferon, Reverse transcription, Integration

## Abstract

**Background:**

SIV_MAC_/HIV-2 Vpx recruits the CUL4A-DCAF1 E3 ubiquitin ligase complex to degrade the deoxynucleotide hydrolase SAMHD1. This increases the concentration of deoxynucleotides available for reverse transcription in myeloid cells and resting T cells. Accordingly, transduction of these cells by SIV_MAC_ requires Vpx. Virus-like particles containing SIV_MAC_ Vpx (Vpx-VLPs) also increase the efficiency of HIV-1 transduction in these cells, and rescue transduction by HIV-1, but not SIV_MAC_, in mature monocyte-derived dendritic cells (MDDCs). Differences in Vpx mechanism noted at that time, along with recent data suggesting that SAMHD1 gains additional restriction capabilities in the presence of type I IFN prompted further examination of the role of Vpx and SAMHD1 in HIV-1 transduction of mature MDDCs.

**Results:**

When challenged with Vpx-VLPs, SAMHD1 was degraded in MDDCs even after cells had been matured with LPS, though there was no increase in deoxynucleotide levels. Steady-state levels of HIV-1 late reverse transcription products in mature MDDCs were increased to the same extent by either Vpx-VLPs or exogenous nucleosides. In contrast, only Vpx-VLPs increased the levels of 2-LTR circles and proviral DNA in myeloid cells. These results demonstrate that exogenous nucleosides and Vpx-VLPs both increase the levels of HIV-1 cDNA in myeloid cells, but only Vpx-VLPs rescue 2-LTR circles and proviral DNA in myeloid cells with a previously established antiviral state. Finally, since trans-acting Vpx-VLPs provide long-lasting rescue of HIV-1 vector transduction in the face of the antiviral state, and exogenous nucleosides do not, exogenous nucleosides were used to achieve efficient transduction of MDDCs by vectors that stably encode Vprs and Vpxs from a collection of primate lentiviruses. Vpr from SIV_DEB_ or SIV_MUS_, Vpx from SIV_MAC251_ or HIV-2, but not SIV_RCM_, degraded endogenous SAMHD1, increased steady-state levels of HIV-1 cDNA, and rescued HIV-1 from the antiviral state in MDDCs.

**Conclusion:**

Inhibition of deoxynucleotide hydrolysis by promoting SAMHD1 degradation is not the only mechanism by which Vpx rescues HIV-1 in MDDCs from the antiviral state. Vpx has an additional effect on HIV-1 transduction of these cells that occurs after completion of reverse transcription and acts independently of deoxynucleotide levels.

## Background

Viruses and their hosts apply selective pressure to each other that influences how each evolves [1,2]. Viruses evolve to escape detection and elimination by the host. In response to changes in the virus there is selection for variations in the host that minimize virus replication or protect against virus-induced pathology. Essential steps in the virus replication cycle in particular offer potential targets for host-encoded viral inhibitors [3]. In the case of retroviruses, reverse transcription of the viral genomic RNA into cDNA, and integration of the viral cDNA into the host cell chromosomal DNA, are essential steps in the viral replication cycle that provide opportunity for the cell to detect and inhibit the virus [4].

Retroviruses from the genus lentivirus, including HIV-1 and at least 40 different lineages of SIVs, infect non-dividing cells such as resting CD4^+^ T cells and myeloid cells. One hallmark of these non-dividing cell types is a concentration of deoxynucleotides (dNTPs) below the threshold required for reverse transcription [5-8]. The cellular enzyme SAMHD1 depletes the intracellular nucleotide pool in non-cycling cells by converting deoxynucleotides into deoxynucleosides and inorganic triphosphates [9]. This depletion of the nucleotide pool inhibits reverse transcription and thereby restricts infection by a range of retroviruses, as well as by some DNA viruses [10-12].

Some SIV lineages, including SIV_SM_, SIV_MAC_, SIV_RCM_, SIV_MND_, as well as HIV-2, encode Vpx, an accessory protein that counteracts SAMHD1 [13-17]. Vpx is not encoded by HIV-1 but shares high similarity to Vpr from which it probably arose by gene duplication [18] or recombination [19]. Though HIV-1 Vpr does not degrade SAMHD1, Vpr from some viruses does have this activity, indicating that the ability to degrade SAMHD1 arose prior to the genesis of Vpx [20]. Like Vpr, Vpx is packaged into the virion via the p6 region of the Gag polyprotein [21,22] where it is required to infect myeloid cells including monocytes, dendritic cells and macrophages [23-29]. Vpx is required for SIV replication in sooty mangabeys, mandrills, and in macaques [30,31].

Via direct interaction with DCAF1, Vpx acts as an adaptor that brings SAMHD1 to the CUL4A E3 ubiquitin ligase complex [32,33]. The result is that SAMHD1 is ubiquitinated and degraded by the proteasome [34]. SAMHD1 degradation is promoted by Vpx from SIV_SM_, SIV_RCM_, and HIV-2, and by Vpr from SIV_MUS_ and SIV_DEB_[20]. The ability to degrade SAMHD1 is species-specific and phylogenetic analysis indicates that the determinants of the Vpr/Vpx interaction with SAMHD1 have been subject to dynamic selective pressure. Vpx from HIV-2 and SIV_MAC_ recognize the C–terminus of SAMHD1 while Vpx from SIV_MND2_ and SIV_RCM_ recognize the SAMHD1 N-terminus [35].

SAMHD1 is phosphorylated in cycling cells and dephosphorylated in IFN-stimulated myeloid cells and resting CD4^+^ T cells [36,37]. Phosphorylation status does not influence deoxynucleotide triphosphohydrolase activity [36-39]. Phosphorylation-defective SAMHD1 mutants retain HIV-1 restriction activity while phosphomimetic mutants lack activity. This suggests that SAMHD1 blocks HIV-1 infection via an additional mechanism that is independent of effects on the nucleotide pool.

Vpx delivery to MDDCs or macrophages with virus like particles (Vpx-VLPs) greatly enhances HIV-1 transduction [40]. Additionally, we have shown in MDDCs [41], and others have confirmed in macrophages [42,43], that Vpx rescues HIV-1 from the antiviral state in IFN-treated MDDCs. Interestingly, Vpx did not rescue SIV_MAC_ or HIV-2 from this antiviral state, and rescue was independent of Vpx binding to DCAF1 [41]. Here we extend these findings by examining the role of SAMHD1 and by comparing the effect of Vpx-VLPs with the effect of exogenous deoxynucleosides; the later intervention overcomes the SAMHD1-mediated block to reverse transcription by increasing the intracellular nucleotide pool. While exogenous deoxynucleosides increased the yield of HIV-1 cDNA in the presence of IFN, Vpx had an additional effect on the level of 2-LTR circles and provirus in monocytes and MDDCs.

## Results

### Vpx-VLPs and exogenous nucleosides rescue HIV-1 from the antiviral state

We have previously shown that the Vpx proteins of SIV_MAC_ and HIV-2 rescue HIV-1 from the antiviral state induced by type I IFN (IFNα/β) or pattern recognition receptor agonists such as LPS, poly(I:C) and poly(dA:dT) [41]. Given that exogenous nucleosides and Vpx-VLPs increase HIV-1 transduction of immature macrophages to a comparable extent [11], the ability of exogenous nucleosides and Vpx-VLPs to rescue HIV-1 transduction of mature MDDCs was compared.

MDDCs were stimulated for 18 hrs with 100 ng/ml LPS to establish the antiviral state. Cells were then treated with SIV_MAC_ Vpx-VLPs produced from SIV3+ and codon optimized Vpx from SIV_MAC251_ expressed *in trans*, to deliver Vpx into the cells, or treated with 2.5 mM deoxynucleosides, to increase the intracellular dNTP pool [11]. After 2 hrs the MDDCs were then challenged with a single cycle HIV-1-GFP reporter virus (NL4-3 GFP) pseudotyped with vesicular stomatitis virus glycoprotein (VSV G).

**Figure 1:**
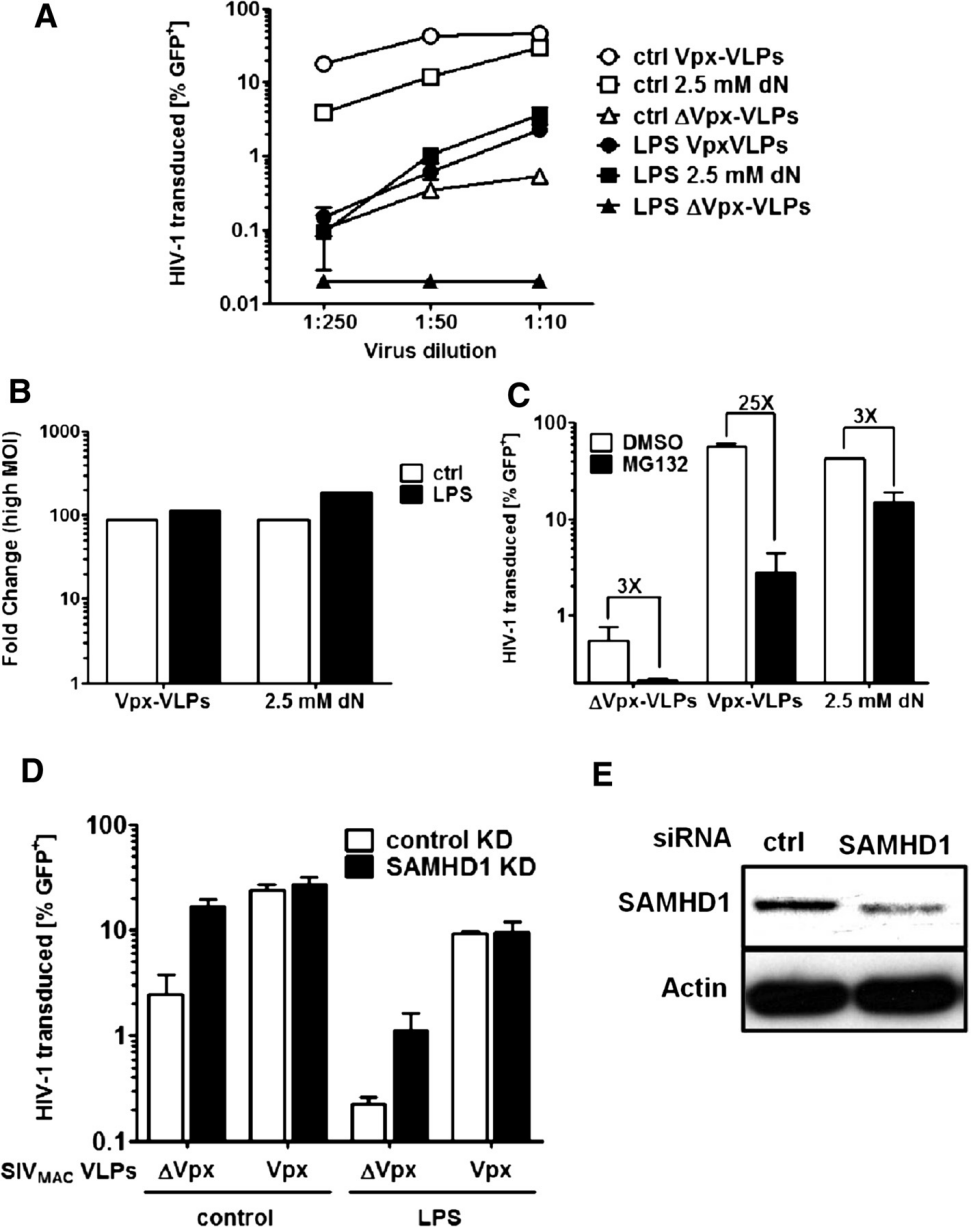
**VpxVLP, exogenous deoxynucleosides and SAMHD1 KD increase HIV-1 infection of MDDC.** MDDCs were stimulated with 100 ng/ml LPS for 18 hrs or not (ctrl), then treated for 2 hrs with either 2.5 mM deoxynucleosides (dN), SIV Vpx-VLPs, or ΔVpx-VLPs, and finally challenged with a VSV G pesudotyped HIV-1 reporter vector (NL4-3 GFP). 3 days later, > 0.5 x 10^5^ out of 0.5 x 10^6^ transduced cells were assessed for GFP expression by flow cytometry, and the detection limit was set to 0.02% (<10 events). Data is displayed as percent GFP-positive cells **(A)** or the higest dilution displayed as fold-change compared to the levels obtained with ΔVpx-VLPs **(B)**. MDDCs were treated for 2 hrs with 1 μg/ml MG132 or DMSO prior to addition of Vpx-VLPs or 2.5 mM deoxynucleosides and challenged with HIV-1 reporter vector 2 hrs later **(C)**. 0.5 x 10^5^ MDDCs were transfected twice with 20 nM siRNA targeting SAMHD1. 6 hrs after the second transfection cells were stimulated for 18 hrs with 100 ng/ml LPS. Cells were then treated for 2 hrs with Vpx-VLPs or ΔVpx-VLPs, challenged with NL4-3 GFP, and analyzed by FACS three days later **(D)**. 0.5 x 10^6^ cells were harvested at the time of challenge with GFP-reporter virus to check SAMHD1 protein levels by western blot **(E)**. All experiments here were repeated on at least three separate occasions with similar results using cells from separate, random, healthy blood donors.

When MDDC were treated with LPS, HIV-1 infection was inhibited to levels below the detection limit (0.02% GFP-positive cells; less than 10 GFP-positive cells in 100,000 cells assessed by flow cytometry) (Figure 1A). Both Vpx-VLPs and nucleoside treatment increased HIV-1 transduction roughly 100-fold above the level of cells treated with SIV_MAC_ VLPs lacking Vpx (ΔVpx-VLPs) (Figure 1B); this was 7-fold above levels of immature MDDCs (no LPS) treated with ΔVpx-VLPs but it did not completely rescue to levels of immature MDDC treated with either Vpx-VLPs or nucleosides (Figure 1A). In immature MDDC (the no LPS condition), Vpx-VLPs and nucleosides increased HIV-1 transduction about 100-fold when compared to immature cells treated with ΔVpx-VLPs. Similar results were obtained in monocyte derived macrophages (MDM) although LPS had a smaller effect on HIV-1 restriction (data not shown). As expected, when cells were treated with the proteasome inhibitor MG132, the Vpx activity was reduced significantly (74-fold) while a much smaller effect (3-fold) was observed on the increased HIV-1 infection with exogenous deoxynucleosides or ΔVpx-VLP treatment (Figure 1C).

To determine the role of SAMHD1 in HIV-1 restriction in the presence of the antiviral state, SAMHD1 levels were reduced in MDDCs by transfecting siRNA targeting SAMHD1 prior to LPS treatment (Figure 1D and 1E). Knockdown of SAMHD1 led to a 7-fold increase in HIV-1 infectivity, which was completely rescued in the control siRNA cells by adding Vpx-VLPs to the cells 2 hrs prior to infection. In the presence of LPS, HIV-1 infectivity was increased 5-fold by SAMHD1 knockdown. Surprisingly, adding Vpx-VLPs rescued HIV-1 from LPS to the same level of infectivity in the SAMHD1 knockdown cells (8-fold) and in the control cells (40-fold) suggesting that Vpx reduced SAMHD1 levels further than the RNAi did or, alternatively, that Vpx overcomes an additional block present in LPS-treated MDDC.

### Vpx degrades SAMHD1 in the presence of the antiviral state but does not increase nucleotide levels

Vpx targets SAMHD1 for degradation by recruiting the E3 ubiquitin ligase complex DCAF1-CUL4A [13,14]. The Vpx Q76A mutant does not bind to DCAF1 and, in the absence of an IFN-induced, antiviral state, this mutant does not increase SIV or HIV-1 transduction efficiency in myeloid cells [33,41].

**Figure 2:**
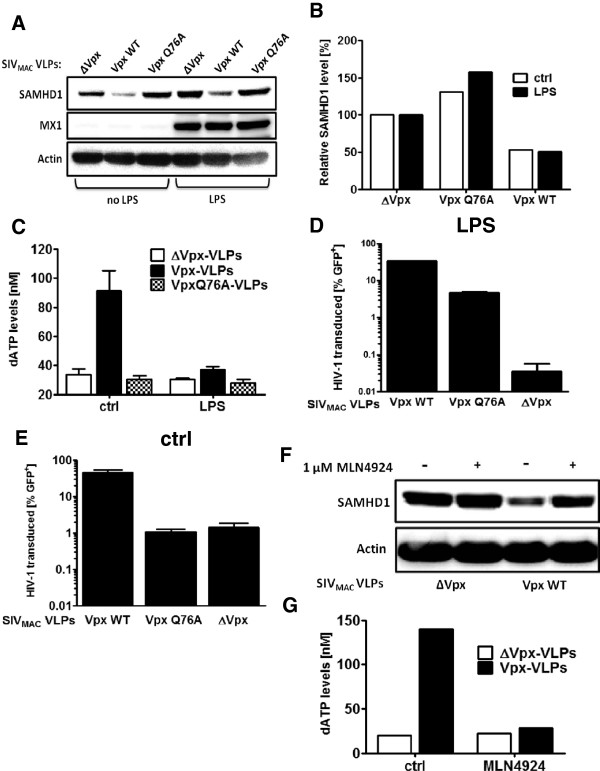
**Vpx degrades SAMHD1 in the presence of the antiviral state but does not increase nucleotide levels.** MDDCs were stimulated with 100 ng/ml LPS for 18 hrs and treated with SIV_MAC_ VLPs containing Vpx, VpxQ76A or no Vpx (ΔVpx) for 24 hrs. 0.8 x 10^5^ cells were harvested for SAMHD1 western blot **(A)**. SAMHD1 protein levels were measured and normalized to the actin signal. The ΔVpx sample was set to 100% **(B)**. 2 x 10^6^ were harvested for nucleotide extraction and measurement of intracellular deoxyadenosine concentration **(C)**. 0.5 x 10^6^ were challenged with NL4-3 GFP after 2 hrs of VLP treatment in the presence of LPS **(D)** or absence **(E)** and 0.5 x 10^5^ were assessed for GFP expression by flow cytometry 3 days later. 1.6 x 10^6^ cells were treated with either 1 μM MLN4924 or DMSO for 2 hrs prior to Vpx-VLPs or ΔVpx-VLPs addition. 24 hrs later cells were harvested for SAMHD1 western blot **(F)** or deoxyadenosine concentration measurement for one sample per condition **(G)**.

To examine the effect of the Vpx Q76A mutant in the context of the antiviral state, MDDC were stimulated with LPS for 18 hrs and then treated for 24 hrs with SIV VLPs containing Vpx or not. Induction of the antiviral state was confirmed by the upregulation of MX1 (Figure 2A), a protein strongly induced by type I IFN [44]. Wild type Vpx reduced SAMHD1 protein levels while the Vpx Q76A mutant did not reduce SAMHD1 protein levels (Figure 2A). SAMHD1 is upregulated in HeLa and HEK 293 cells by type I IFN ([45] and data not shown). There was only a small increase of 20% in SAMHD1 levels by LPS and protein levels were reduced to below 50% in absence and presence of LPS (Figure 2B and Additional file 1: Figure S1 and Additional file 2: Figure S2). The reduction of SAMHD1 protein levels coincided with an increase in intracellular deoxynucleotide levels as shown by intracellular deoxyadenosine concentration (Figure 2B and Additional file 1: Figure S1 and Additional file 2: Figure S2). Nucleotide measurements were performed in the linear range of the assay (4–128 fmol) and samples were diluted if necessary. The volume of dendritic cells to calculate the intracellular dNTP concentration was determined to be 1000 μm^3^ as previously shown [46]. The detection threshold for the assay was determined to be at 20 nM for dATP. Surprisingly, SAMHD1 protein levels were reduced by Vpx in the presence of LPS, but the nucleotide concentration remained at the level of ΔVpx-VLPs and Vpx Q76A. The nucleotide concentration in MDDCs was 20–40 nM, well below the HIV-1 RT K_m_ (>100 nM) [6,47]. Despite these low nucleotide levels, Vpx and Vpx Q76A were still able to rescue HIV-1 from the antiviral state 971-fold and 137-fold, respectively (Figure 2D). In the absence of the antiviral state Vpx Q76 was not able to increase HIV-1 (Figure 2E).

### CUL4A inhibitor MLN4924 blocks Vpx induced SAMHD1

A covalent modification by NEDD8 of CUL4A and other cullin-RING E3 ligases (CRL) is required for their ubiquitin ligase activity [48]. The pharmacological inhibitor MLN4924 inhibits the DCAF1-CUL4A E3 ligase complex by blocking the NEDD8 activation enzyme (NAE). It has been shown that MLN4924 inhibits the degradation of APOBEC3G (A3G) by the HIV-1 accessory protein Vif, which uses CUL5 to target A3G for proteasomal degradation [49]. MDDCs were treated for two hours with 1 μM of MLN4924 and then Vpx-VLPs or ΔVpx-VLPs were added. In the presence of MLN4924 Vpx was no longer able to induce degradation of SAMHD1 (Figure 2E) and nucleotide levels remained low in MLN4924 treated samples (Figure 2F). The same was observed using proteasome inhibitor MG132 (data not shown). Due to toxicity, infectivity assays could not be performed in cells treated with MLN4924 or MG132 in combination with LPS.

### Exogenous nucleosides increase SIV infection but do not rescue SIV from the antiviral state

By degrading SAMHD1, Vpx increases the available nucleotide pool for reverse transcription which increases the infectivity of a range of retroviruses [10]. We have previously shown that while Vpx rescues HIV-1 100 to 1000-fold from the IFN-induced antiviral state, it does not rescue SIV_MAC_ transduction in this context [41]. To compare the effect of exogenous deoxynucleosides on SIV_MAC_ and HIV-1, MDDCs were stimulated with 3 mM deoxynucleosides for 2 hrs and then infected with an HIV-1-GFP reporter vector or a SIV_MAC-_GFP reporter vector, deleted for Vpx or not. All vectors were pseudotyped with VSV G and normalized for titer by titration on HeLa cells.

**Figure 3:**
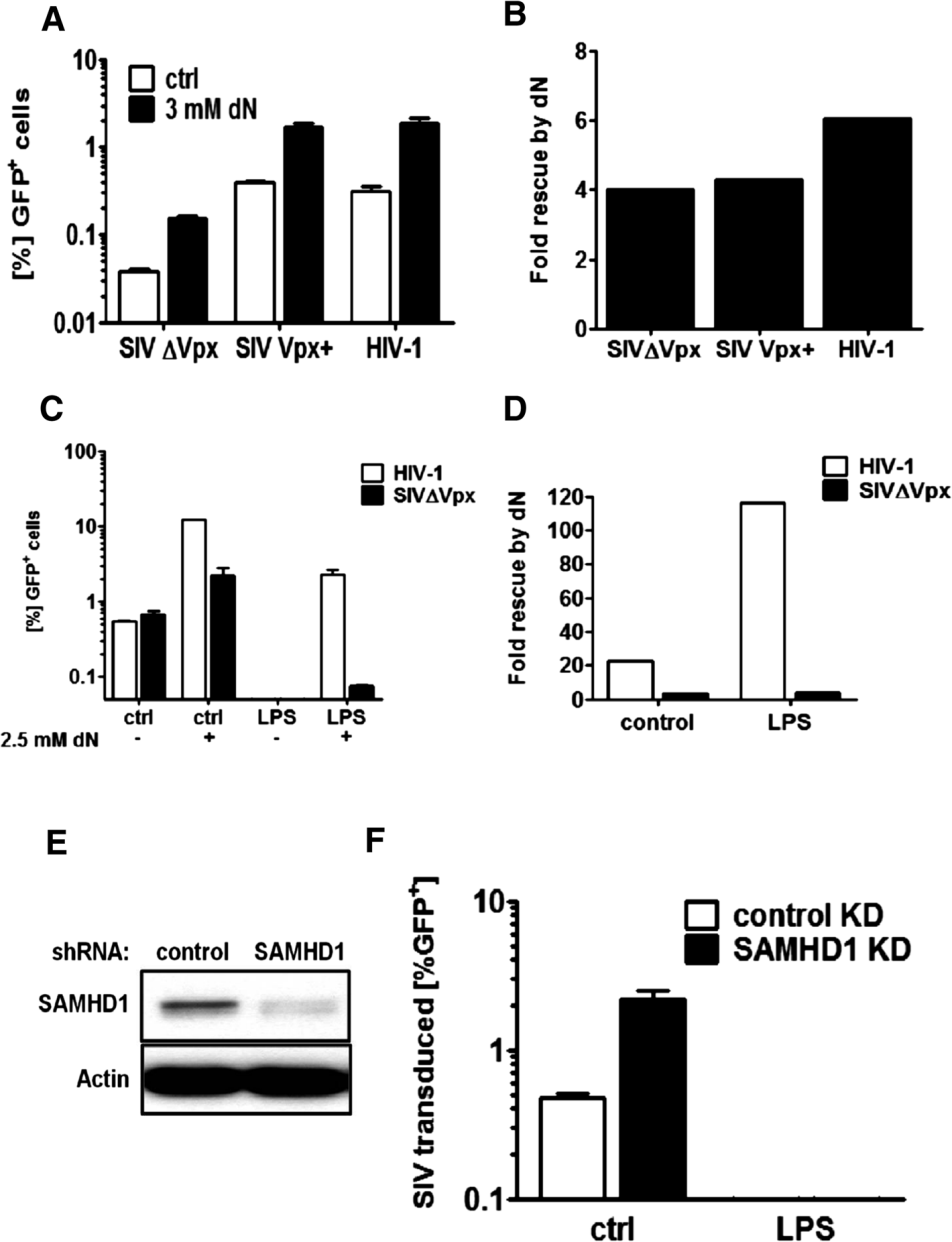
**Exogenous deoxynucleosides and SAMHD1 KD do not rescue SIV from the antiviral state.** MDDCs were treated for 3 hrs with 3 mM deoxynucleosides (dN) and either challenged with a VSV G pseudotyped SIV-GFP reporter vector deleted for Vpx (SIVΔVpx) or not (SIV Vpx+) or with an HIV-1-GFP reporter vector. The viruses were normalized by titration on HeLa cells. Values are displayed in %GFP positive cells **(A)** or fold rescue by exogenous deoxynucleosides **(B)**. MDDCs were stimulated with 100 ng/ml LPS for 18 hrs, treated with 2.5 mM deoxynucleosides (dN) for 2 hrs and challenged with a SIVΔVpx or HIV-1 reporter vector. Values are displayed in %GFP positive cells **(C)** or fold-rescue by exogenous deoxynucleosides **(D)**. To achieve SAMHD1 knockdown, freshly isolated CD14^+^ monocytes were treated for 2 hrs with Vpx-VLPs and then transduced with a lentiviral vector expressing shRNA targeting SAMHD1 (SAMHD1 KD) or luciferase (control KD) and differentiated into MDDCs over 5 days. 0.5 × 10^6^ were used to assess knockdown efficency by western blot **(E)**. Knockdown cells were stimulated with 100 ng/ml LPS and challenged with a Vpx + SIV GFP reporter vector (SIV_MAC239_) **(F)**.

Addition of exogenous deoxynucleosides increased the infectivity of all three vectors from 4 to 6-fold (Figure 3A and 3B), although, as expected, absolute infectivity was lower for SIVΔVpx than for the other two viruses (Figure 3A). To test if exogenous nucleosides rescued SIV_MAC_ from the antiviral state as effectively as they rescued HIV-1, MDDC were stimulated with LPS for 18 hrs and then treated with nucleosides for 2 hrs prior to infection with HIV-1 or SIVΔVpx. Nucleosides rescued HIV-1 over 100-fold from the antiviral state but they did not rescue SIV_MAC_ (Figure 3C and 3D). We have previously shown that SIV expressing Vpx is not rescued by from the antiviral state by Vpx-VLPs [41].

To determine if SAMHD1 degradation rescues SIV from the antiviral state, SAMHD1 was knocked down using shRNA. Monocytes were treated with Vpx-VLPs after isolation from donor blood and transduced with a lentiviral vector expressing a puromycin resistance cassette and a shRNA targeting SAMHD1 in a miR-30 backbone [41,50,51]. After differentiation into MDDCs with IL4 and GM-CSF, the cells were stimulated for 18 hrs with LPS to induce an antiviral state and then challenged with a SIV-GFP reporter vector expressing Vpx. Using Vpx-VLPs to transduce monocytes with a knockdown vector decreased SAMHD1 levels greatly (Figure 3E). Knockdown of SAMHD1 increased SIV infectivity 4-fold in the absence of LPS but, in the presence of LPS, SIV infection could not be detected (Figure 3F). These results suggest that SIV is blocked in cells in the antiviral state by a factor different from SAMHD1 and not targetable by Vpx.

### Vpx increases HIV-1 2-LTR circles in LPS-treated MDDCs

To compare the effect of exogenous deoxynucleosides with the effect of Vpx on HIV-1 reverse transcripts and metabolites, MDDC were stimulated with LPS for 18 hrs, incubated with either Vpx-VLPs (Vpx) or 2.5 mM deoxynucleosides alone (dN), or with the two in combination (dN + Vpx), for 2 hrs and then infected with an VSV G-pseudotyped, HIV-1-GFP reporter vector, or with a control vector lacking the VSV G protein (ΔVSVg). After 24 hrs, low molecular weight DNA was harvested and real-time PCR for full length HIV-1 cDNA (LRT) and 2-LTR-circles was performed. HIV-1 cDNA levels were normalized to host cell mitochondrial DNA and compared to cDNA levels from cells treated with ΔVpx-VLPs.

**Figure 4:**
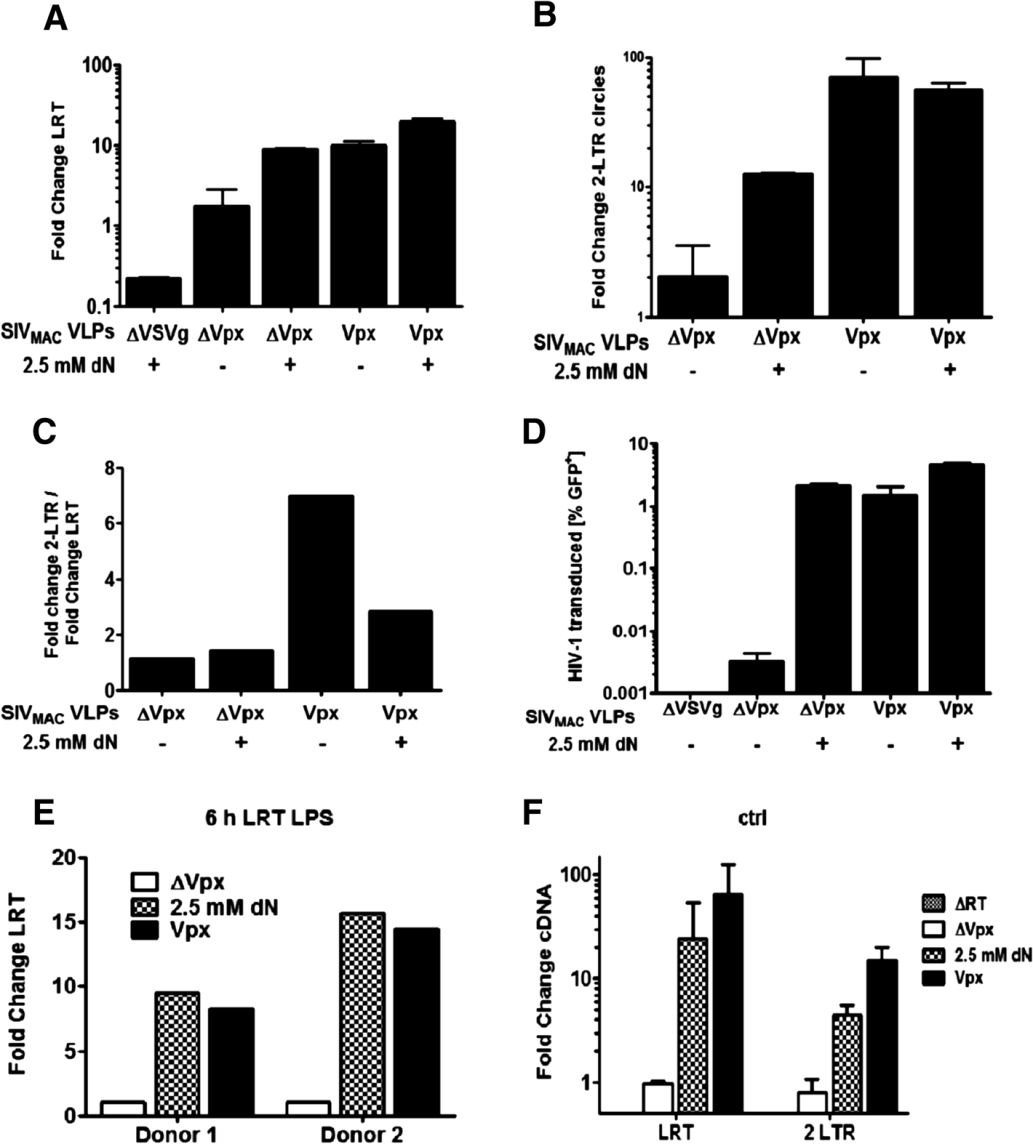
**Vpx increases HIV-1 2-LTR circles in LPS-treated MDDCs.** 2 × 10^6^ MDDCs were stimulated with 100 ng/ml LPS for 18 hrs, treated for 2 hrs with either ΔVpx-VLPs (ΔVpx) or Vpx-VLPs alone or combined with 2.5 mM deoxynucleosides (dN) and infected with NL4-3 GFP or NL4-3 GFP lacking VSV G protein (ΔVSVg) as negative control. 24 hrs later low molecular weight DNA was isolated and real-time PCR for full length HIV-1 cDNA (LRT) **(A)** or 2-LTR circles **(B)** was performed and the ratio of fold change of 2-LTR circles over LTR was displayed **(C)**. LRT and 2-LTR circles were normalized to mitochondrial DNA and compared to ΔVpx levels. GFP positve cells were assessed for GFP expression by flow cytometry after 72 hrs **(D)**. MDDC from two donors were stimualted with 100 ng/ml LPS, treated with either ΔVpx-VLPs, Vpx-VLPs or 2.5 mM deoxynucleosides and infected 3 hrs later. Low molecular DNA from single samples was isolated 6 hrs p.i. and real-time PCR for full length HIV-1 cDNA was prefromed **(E)**. MDDC were treated with either ΔVpx-VLPs, Vpx-VLPs or 2.5 mM deoxynucleosides and infected 3 hrs later. Low molecular DNA from single samples was isolated 12 hrs p.i. for real-time PCR for full length HIV-1 cDNA and 24 hrs p.i for 2-LTR cirlce real-time PCR **(F)**.

Exogenous deoxynucleosides, as well as Vpx-treatment, increased LRT products 10-fold measured at 24 hrs (Figure 4A) as well as at 6 hrs (Figure 4E). There was little variation among different donors, with the lowest donor showing a 4-fold increase. In the absence of the VSV G, or using a HIV-1-GFP reporter vector with the reverse transcriptase active site mutation D185K/D186L, LRT products were nearly undetectable (Figure 4A and Figure 4F), indicating that PCR signals were not due to carry-over of plasmid DNA from the transfection that produced the vectors. In the absence of the antiviral state both Vpx-VLPs treatment and exogenous nucleosides increased LRT cDNA levels measured at 12 hrs post infection (Figure 4F).

2-LTR circles are used as a marker for nuclear import of retroviral cDNA since they are believed to be created by end-to-end ligation of the two LTRs by cellular enzymes in the nucleus. The level of 2-LTR circles was measured using primers in which one anneals to the 2-LTR junction; this precludes detection of autointegration events that are detected by primers flanking the junction [52]. Treating MDDCs with deoxynucleosides increased 2-LTR circles about 10-fold, as compared to levels in cells treated with control VLPs that lack Vpx (Figure 4B). Interestingly, treatment of cells with Vpx-VLPs increased 2-LTR circles 40- to 70-fold. Unfortunately, provirus was not detectable by Alu-PCR in MDDCs that had been treated with LPS, even 72 hrs after infection. As previously observed (Figure 1A) infectivity levels did not differ after treatment with either Vpx-VLPs or exogenous nucleosides alone or in combination (Figure 4D). In the absence of the antiviral state Vpx-VLPs treatment increased 2-LTR circle formation 3-fold higher compared to exogenous nucleosides (Figure 4F), indicating that this effect of Vpx is also be observed to some extent in immature DCs. This could explain the higher infectivity levels observed in Figure 1A. These results show that Vpx-VLPs increase HIV-1 2-LTR circle formation in LPS-treated MDDCs, an effect that was not observed in cells that were treated with exogenous deoxynucleosides.

### Vpx increases the HIV-1 provirus content in monocytes

**Figure 5:**
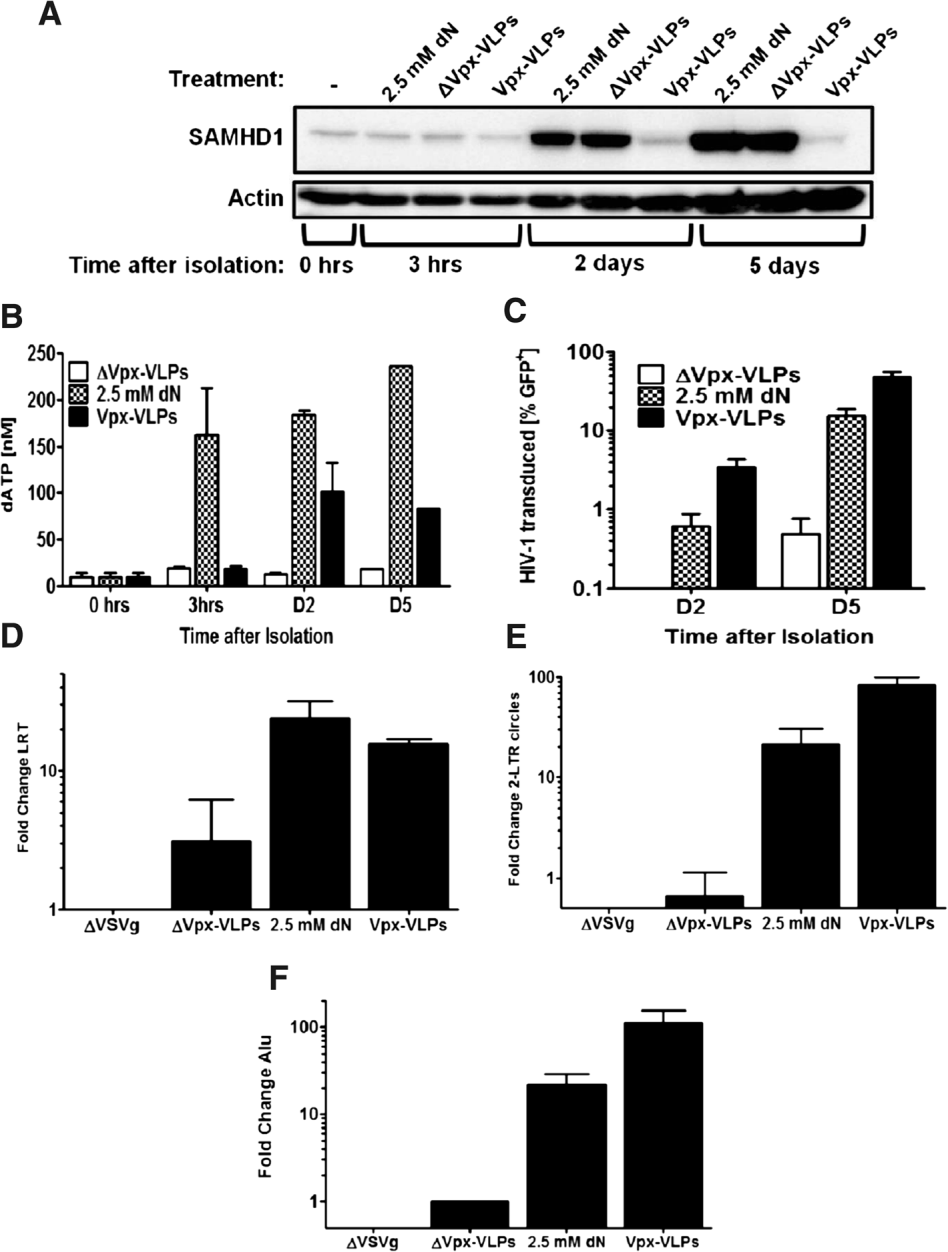
**Vpx increases HIV-1 provirus content in monocytes.** Freshly isolated CD14^+^ monocytes from human peripheral blood were either treated with ΔVpx-VLPs, Vpx-VLPs or 2.5 mM deoxynucleosides. 1 × 10^6^ cells were harvested for western blot **(A)**, 2 × 10^6^ collected for nucleotide measurement **(B)** and 0.5 × 10^6^ were challenged 2 hrs after treatment with a HIV-1 reporter vector to assess GFP expression by flow cytometry **(C)**. Samples were collected before treatment (0 hrs), 3 hrs two days (D2) and five days (D5) after treatment. HIV-1 LRT **(D)** and 2-LTR circles **(E)** were measured 3 days after infection and integrated provirus was analyzed by Alu-PCR at day 5 **(F)**.

**Figure 6:**
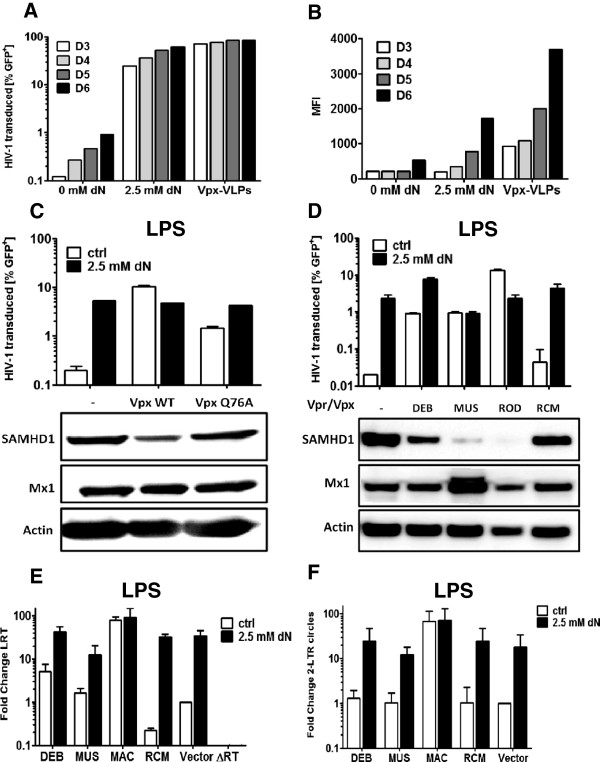
**Transduction of MDDCs with lentivectors expressing SIV Vpx or Vpr.** Freshly isolated CD14^+^ monocytes from human peripheral blood were treated with either Vpx-VLPs or 2.5 mM deoxynucleosides and transduced with a lentiviral vector expressing GFP. Cells were analyzed over the course of differentiation (day 3 to day 6) for GFP expression by flow cytometry shown as percent GFP-postitve cells **(A)** or mean fluoresence intensity **(B)**. Monocytes were treated with 2.5 mM deoxynucleosides for 2 hrs and transduced with a lentiviral vector expressing a puromycin-resistance cassette alone (−), or with the indicated SIV_MAC_ Vpx WT or Vpx Q76A mutant **(C)**, Vpr from SIV_DEB_ (DEB), Vpr from SIV_MUS_ (MUS), Vpx from HIV-2 (ROD), Vpx from SIV_RCM_ (RCM) or the empty vector (Vector) **(D)**. Cells were selected with 1 μg/ml puromycin for 24 hrs at day 3 after transduction. After selection cells were harvested, washed, re-plated and stimulated with 100 ng/ml LPS for 18 hrs. Cells were treated or not with 2.5 mM deoxynucleosides for 2 hrs and challenged with HIV-1-GFP reporter vector. GFP positve cells were assessed for GFP expression by flow cytometry 72 hrs later. Low molecular weight DNA was collected at 24 hrs after challenge and LRT **(E)** and 2 LTR circles **(F)** were measured by qPCR.

Treatment of monocytes with Vpx-VLPs allows transduction of these cells with lentiviral vectors and reporter viruses [51]. To investigate the effect of exogenous deoxynucleosides on monocytes, freshly isolated CD14^+^ cells were treated with either 2.5 mM deoxynucleosides (dN), SIV Vpx-VLPs, or ΔVpx-VLPs over the entire duration of the experiment. Protein samples for western blot analysis and cellular nucleotide extraction were taken before treatment (0 hrs), at 3 hrs, two days (D2), and five days (D5) after treatment with deoxynucleosides or VLPs. Over the duration of the monocyte differentiation into DCs, SAMHD1 levels increased considerably in the cells treated with VLPs lacking Vpx or nucleosides while SAMHD1 levels were nearly undetectable in cells treated with Vpx-VLPs (Figure 5A). The intracellular nucleotide concentration was increased 10-fold in cells treated with Vpx-VLPs at day 2 after treatment and remained at the same level also at day 5. Treatment with deoxynucleosides led to a rapid increase in cellular nucleotide concentration already 3 hrs after treatment and increased until day 5 (Figure 5B). Treatment with either Vpx-VLPs or nucleosides increased infection with a HIV-1 GFP reporter vector (Figure 5C). Vpx-VLP treatment increased HIV-1 infection 3-fold more than nucleosides (48% compared to 15% GFP-positive cells). LRT and 2-LTR products were detected three days after infection. LRT levels were very low but increased 5 and 7.5-fold by Vpx-VLPs and exogenous nucleosides, respectively (Figure 5D). 2-LTR circles were increased about 20-fold by nucleosides while Vpx-VLP-treated samples showed an 80-fold increase compared to cells treated with ΔVpx-VLPs (Figure 5E). This increase in 2-LTR circles was associated with an increase in provirus. While nucleoside treatment led to a 20-fold increase compared to ΔVpx-VLPs treatment, Vpx-VLP-treatment increased provirus levels 110-fold (Figure 5F). The increased provirus levels were also reflected in an increase in mean fluorescence intensity being 2.5–fold higher in the Vpx-VLPs treated cells (Figure 6B). This is consistent with the finding above that, relative to exogenous deoxynucleosides, Vpx-VLPs increase the levels of provirus leading to the increased expression of GFP, which results in the difference of MFI measured (Figure 6B).

These results indicate that similar to MDDCs, deoxynucleosides are able to overcome the SAMHD1 block to reverse transcription and also increase 2-LTR circles formation and provirus integration but Vpx-VLPs treatment results in 4 to 5-fold higher 2-LTR circle and provirus levels compared to nucleosides.

### Transduction of MDDCs with lentivectors expressing SIV Vpx or Vpr

To determine if Vpx and Vpr proteins encoded by different SIVs have the capacity to degrade endogenous SAMHD1 in MDDCs and to rescue HIV-1 from the antiviral state in these cells, MDDCs were stably transduced with lentivectors that express the different Vpx and Vpr proteins. Since pre-treating monocytes with Vpx-VLPs leads to a permanent decrease of SAMHD1 (Figure 5A), this approach could not be used to increase transduction rate of HIV-1 vectors that stably express these proteins. To test if treatment with exogenous deoxynucleosides boosts monocyte transduction by lentiviral vectors, freshly isolated monocytes were treated with either 2.5 mM deoxynucleosides or Vpx-VLPs for 2 hrs without removal over the duration of the differentiation and transduced with a lentiviral vector encoding a GFP cassette and a puromycin selection cassette under the control of two independent promoters [53]. Although treatment with exogenous nucleosides or Vpx-VLPs increased MDDC transduction (Figure 6A) to similar levels (62% vs. 85%) mean fluorescence intensity was 2.5–fold higher in the Vpx-VLPs treated cells (Figure 6B). This is consistent with the finding above that, relative to exogenous deoxynucleosides, Vpx-VLPs increase the levels of provirus (Figure 5F).

Expression levels of lentivectors transduced in the presence of exogenous nucleosides was not sufficient to achieve knockdown of SAMHD1 or other genes, or to detect Vpx protein after transduction with a lentiviral vector encoding Vpx and a puromycin selection cassette (data not shown). Yet, these transduction conditions were sufficient to express *vpx* in MDDCs using a lentiviral vector encoding both Vpx and a puromycin selection cassette (Figure 6C). Nonetheless, in MDDCs transduced with an SIV_MAC_ vpx-expression vector and treated with LPS, SAMHD1 was degraded and HIV-1 transduction was rescued 50-fold. As expected, Vpx Q76A did not degrade SAMHD1 but still rescued HIV-1 7-fold from the antiviral state. Unlike the permanent effect of Vpx-VLPs, the effect of deoxynucleosides used for lentiviral vector transduction at the monocyte stage disappeared after removing the cells from medium supplemented with nucleosides (compare Figure 5B), since HIV-1 was blocked by LPS and rescued 26-fold by re-addition of deoxynucleosides.

The same approach was used to express Vpr encoded by SIV_DEB_ and SIV_MUS_, and Vpx from SIV_RCM_ and HIV-2. HIV-2_ROD_ Vpx and both SIV_DEB_ SIV_MUS_ Vpr are reported to be able to degrade human SAMHD1 [20] while SIV_RCM_ Vpx only degrades SAMHD1 from red capped mangabey. As expected SIV_RCM_ Vpx was not able to degrade human SAMHD1 (Figure 6D) and was not able to rescue HIV-1 from the antiviral state. SIV_DEB_ and SIV_MUS_ Vpr on the other hand were able to increase HIV-1 transduction efficiency 45-fold in the face of an antiviral state. SIV_MUS_ Vpr almost completely degraded SAMHD1 while Vpr from SIV_DEB_ was less efficient. This might be due to expression levels being lower for SIV_DEB_ Vpr than for SIV_MUS_ when compared in transfected HEK 293 cells with HA-tagged Vpr/Vpx (data not shown). Lower expression level might also explain why adding deoxynucleosides increased HIV-1 transduction in the SIV_DEB_ Vpr cells while there was no additional effect observed in the SIV_MUS_ Vpr cells. Both Vpr from SIV_DEB_ and SIV_MUS_ increased HIV-1 LRT compared to cells transduced with the Vpx from SIV_RCM_ of 23-fold and 7-fold, respectively. Nucleosides further increased LRT product 190-fold in SIV_DEB_ Vpr and 54-fold in SIV_MUS_ Vpr transduced cells above SIV_RCM_ Vpx levels without nucleosides. Vpx from SIV_MAC_ had the greatest effect on LRT levels (350-fold) and nucleosides only a small effect (Figure 6E). Unexpectedly, Vpr from SIV_DEB_ and SIV_MUS_ only had a small effect on 2-LTR circle levels (3-fold increase) which was enhanced further by the addition of deoxynucleosides. SIV_MAC_ Vpx led to an increase of 135-fold with no additional effect of nucleosides, confirming the results from Figure 4B. In the presence of SIV_MAC_ Vpx 2-LTR circles were increased 4-fold compared to cells expressing the inactive Vpx from SIV_RCM_ treated with exogenous nucleosides. Although Vpr from SIV_DEB_ and SIV_MUS_ increased HIV-1 LRT, only Vpx from SIV_MAC_ was able to increase 2-LTR circles, indicating that this might be a conserved function of the Vpx of the SIV_SM_/HIV-2 lineage.

## Discussion

Exogenous deoxynucleosides and Vpx-VLPs both increase the efficiency of HIV-1 transduction. At first glance, both interventions appear to rescue HIV-1 via similar mechanisms. Exogenous deoxynucleotides are taken up by DCs and this presumably replenishes the dNTPs that are maintained at low level by SAMHD1 in these cells (Figure 5B). Vpx-VLPs degrade SAMHD1 and thereby prevent dNTP hydrolysis. In the context of a type I IFN-mediated antiviral state in MDDCs, though, this is not the only mode of action by which Vpx rescues HIV-1. We have previously shown that Vpx is able to rescue HIV-1 but not SIV_MAC_ or HIV-2 from the antiviral state induced by type I IFN itself (IFNα/β) or PRR agonists such as LPS, poly(I:C) and polydAdT [41]. Here we extend these findings to the HIV-1 restriction factor SAMHD1.

SAMHD1 restricts HIV-1 replication in resting CD4^+^ T cells [16,17] and myeloid cells [13-15] by depleting the intracellular nucleotide pool [11]. The nucleotide concentration in macrophages is 20 to 40 nM, well below the HIV-1 RT K_m_ of >100 nM, and orders of magnitude lower than the 2 to 5 μM measured in cycling CD4^+^ T cells [6,47]. Dendritic cells have levels similar to those of macrophages (Figure 2B) and these levels do not change upon LPS stimulation [45]. As shown by others in macrophages [11,42,43], and here by us in DCs (Figure 1A and Figure 3C) and monocytes (Figure 5), addition of exogenous deoxynucleosides artificially increases the intracellular deoxynucleotide pool, allowing HIV-1 reverse transcription to take place [11].

It has been reported that in macrophages Vpx does not rescue HIV-1 from IFNα treatment despite SAMHD1 degradation and increased nucleotide levels [42,43]. In these reports Vpx-VLPs or the addition of exogenous deoxynucleosides increased HIV-1 infectivity to the level seen with no Vpx treatment in the absence of the antiviral state. In DCs we observed an increase of HIV-1 infection in absence and presence of the antiviral state of about 100-fold (Figure 1A and Figure 1B) which coincides with Vpx and nucleoside having a similar effect on HIV-1 full length cDNA (LRT) of about 10-fold increase (Figure 4A) in the presence of the antiviral state as well as in the absence (Figure 4F) and [5,45]. When cells were treated with LPS neither deoxynucleosides nor Vpx-VLPs were able to fully rescue HIV-1 infectivity to the level of Vpx and deoxynucleosides without LPS indicating that there is indeed a Vpx- and SAMHD1-independent factor upregulated in the antiviral state.

While in type I IFN stimulated macrophages SAMHD1 degradation led to an increase in intracellular deoxynucleotide levels [42,43], this was not observed in MDDCs (Figure 2A). It has been shown that SAMHD1 is protected from Vpx mediated degradation in primary myeloid and plasmacytoid dendritic cells [54] and in type I IFN treated THP-1 cells [43]. Although we see a small increase in SAMHD1 levels in LPS treated MDDCs, SAMHD1 is degraded to similar levels in the presence of the antiviral state (Figure 2B and Additional file 1: Figure S1 and Additional file 2: Figure S2). This could indicate that the regulation of the intracellular deoxynucleotide concentration in MDDCs occurs at multiple levels and results from a balance of deoxynucleotide degradation by SAMHD1 and newly produced deoxynucleotides by such enzymes as ribonucleotide reductase [55].

Interestingly, as we have previously reported, Vpx does not rescue SIV_MAC_ or HIV-2 from the antiviral state in MDDCs. The factor blocking SIV in MDDCs in the antiviral state is independent of SAMHD1 and independent of Vpx (Figure 3) since neither SAMHD1 knockdown, nor addition of exogenous nucleosides, nor Vpx-VLPs were able to rescue SIV from LPS [10,38]. It could be that the factor that flattens SIV_MAC_ in MDDCs in the antiviral state is the same factor that inhibits HIV-1.

SAMHD1 knockdown in MDDCs using siRNA increased HIV-1 levels in the absence of LPS and also, to a smaller extent, in the presence of LPS. SAMHD1 knockdown in THP-1 cells is reported to increase HIV-1 infectivity by boosting intracellular dNTP levels [11]. In MDDCs, Vpx-VLPs increased HIV-1 infectivity even after SAMHD1 knockdown (Figure 1D). This could be because Vpx decreases SAMHD1 levels beyond that of the knockdown which would then further increase the dNTP pool. Alternatively, Vpx-VLPs might remove an additional block present in LPS-treated MDDCs that acts independently of nucleotide levels. The latter explanation is supported by the observation that, despite SAMD1 degradation and a 100-fold rescue of HIV-1 infectivity, there was no increase in nucleotide levels by Vpx-VLPs in LPS-treated MDDCs (Figure 2B).

Our findings are in accord with recent reports that the constitutive deoxynucleotide triphosphorylase activity of SAMHD1 is distinct from its retroviral restriction activity, which is regulated by phosphorylation [36-38]. SAMHD1 is phosphorylated at threonine 592 in dividing, HIV-1 permissive cells, such as activated T cells or cycling THP-1 cells. SAMHD1 is dephosphorylated in HIV-1 non-permissive cells such as PMA-arrested THP-1 cells or monocytes. In macrophages and MDDCs a portion of SAMHD1 is phosphorylated, and this residual phosphorylation disappears with type I IFN treatment, suggesting the presence of an IFN-inducible phosphatase. Vpx degrades SAMHD1 independent of its phosphorylation status [36].

The block to HIV-1 reverse transcription that results from SAMHD1-mediated depletion of dNTP can be overcome by either Vpx-VLPs or by exogenous nucleosides. This was shown by the fact that both Vpx and exogenous deoxynucleosides increase HIV-1 cDNA (Figure 4A). But there was an additional block in LPS-treated myeloid cells, at the level of 2-LTR circles and provirus establishment that was overcome by Vpx-VLPs, but not by exogenous deoxynucleosides (Figure 4B). Vpx-VLPs, but not exogenous deoxynucleosides, remove SAMHD1 (Figures 2A and 6C), which is de-phosphorylated in MDDCs under these conditions [36]. The post-reverse transcription block that occurs with LPS treatment might be due to SAMHD1 and its recently described ability to bind HIV-1 cDNA and to cleave single-stranded RNA and DNA [56]. Despite the fact that exogenous nucleosides had a greater effect on cellular nucleotide levels than did Vpx-VLPs in monocytes (Figure 5B), Vpx increased HIV-1 infection to higher levels than did exogenous deoxynucleosides (Figure 5C). This increase in infectivity correlated with the fact that Vpx-VLPs increased 2-LTR circle levels 4-fold higher, and provirus levels 5-fold higher, than with nucleosides (Figure 5E and Figure 5F).

We and others have used Vpx-VLPs to achieve lentiviral transduction levels sufficient to knockdown genes in MDDCs [41,50,51,57]. While this technique works well, it has to be considered that monocytes treated with Vpx-VLPs still do not express SAMHD1 5 days after differentiation into MDDCs (Figure 5A). Treating monocytes with deoxynucleosides did not change SAMHD1 levels (Figure 5A) but this permits lentiviral vector transduction, although at a lower level than when monocytes are treated with Vpx-VLPs. Using exogenous deoxynucleosides, Vpx and the Q76A Vpx mutant were expressed from within MDDCs, without prior degradation of SAMHD1 by Vpx-VLPs. As expected the Vpx Q76A mutant was unable to degrade SAMHD1 and it failed to increase dNTP levels (Figures 2B and 6C). Nonetheless, Vpx Q76A was able rescue HIV-1 from the antiviral state (Figure 6C), indicating yet again that the boost to HIV-1 infectivity is not all due to effects on deoxynucleotide levels.

SAMHD1 dNTP depletion activity requires tetramerization [58]. Binding of Vpx to SAMHD1, and subsequent recruitment of the CUL4A-DCAF complex disrupts SAMHD1 activity before its degradation by the proteasome [59]. Given these observations, and data presented here (Figure 2A-D) and previously [41], our data are consistent with a model in which the Vpx Q76A mutant retains the ability to bind to SAMHD1, but the mutant does not interact with DCAF1 and therefore does not recruit CUL4A. SAMHD1 is therefore not degraded, but the mutant Vpx might still interfere with SAMHD1 oligomerization. This would explain how the mutant could have no effect on dNTP levels yet still provide significant rescue of HIV-1 transduction in LPS-treated MDDCs. Another possibility is that there is an additional block to HIV-1 in LPS-treated MDDCs that is altogether independent of SAMHD1 and the DCAF1-CUL4A complex.

The Vprs encoded by SIV_DEB_ and SIV_MUS_ have been reported to degrade SAMHD1 from certain species, including human SAMHD1 [20]. Vpx from HIV-2 or SIV_MAC_, and Vpr from SIV_MUS_, each recognize the C-terminus of SAMHD1, while Vpx from SIV_MND2_ and SIV_RCM_ recognize the N-terminus of SAMHD1. Vpr from SIV_DEB_ recognizes both the C- and N-termini of SAMHD1 and thus targets the broadest range of SAMHD1 orthologues [35]. Here we exploited the increased lentivector transduction with exogenous deoxynucleosides to show for the first time that these Vpr proteins are able to degrade endogenous human SAMHD1 in MDDCs and increase the levels of HIV-1 cDNA and infectivity. Compared to Vpx from SIV_MAC,_ Vpr from SIV_MUS_ and SIV_DEB_ do not increase HIV-1 2-LTR levels suggesting that Vpx from the SIV_SM_/HIV-2 lineage might possess an additional activity which is not conserved in all SIV Vpr/Vpx genes able to degrade SAMHD1.

## Conclusions

Here we showed that Vpx removes SAMHD1 protein and rescues HIV-1 transduction from the antiviral state in LPS-treated MDDCs, largely independently of effects on intracellular deoxynucleotide levels. Vpx increases the level of 2-LTR circles and provirus in myeloid cells, an effect which was not observed by artificially increasing the intracellular nucleotide pool with exogenous nucleosides.

## Methods

### Ethics statement

Buffy-coats obtained from anonymous blood donors were provided by the Blood Transfusion Center of the Hematology Service of the University Hospital of Geneva by agreement with the service, after approval of our project by the Ethics Committee of the University Hospital of Geneva (Ref #0704).

### Cell lines, primary cells, cytokines, and tissue culture

HEK 293 cells were provided by Dr. Walter Mothes (Yale University) and HEK 293FT cells were obtained from Invitrogen. HEK 293, HeLa, and HEK 293FT cells were grown in Dulbecco’s modified Eagle medium (D-MEM) (high glucose) with 10% FBS (PAA), 20 mM L-glutamine, and 1000 mg/ml Penicillin-Streptomycin (Invitrogen).

Peripheral blood mononuclear cells (PBMC) were isolated from healthy donor buffy coats using Ficoll-Paque Plus (GE Healthcare). CD14^+^ monocytes were enriched from PBMC by positive selection using CD14 MicroBeads following the manufacturer’s protocol (Miltenyi Biotec). Enrichment was routinely verified to be greater than 95% using phycoerythrin (PE)-conjugated monoclonal antibodies against CD14. CD14^+^-enriched cell populations were counted, centrifuged at 200 × g for 10 min, and resuspended at 2 × 10^6^ cells/ml in RPMI-1640 supplemented with 10% FBS (Hyclone), 1× MEM NEAA, 20 mM L-glutamine, 25 mM HEPES, 1000 mg/ml penicillin-streptomycin, 1 mM sodium-pyruvate and 50 μM β-Mercaptoethanol (all from GIBCO). To induce differentiation of monocytes into dendritic cells (MDDCs), recombinant human granulocyte-macrophage colony-stimulating factor (GM-CSF) and recombinant human interleukin 4 (IL-4) conditioned medium was added at a dilution of 1:50 and cells were cultured for 5 days at 37°C in 5% CO_2_.

Cytokine-conditioned medium was produced in HEK 293 cells transduced with a lentiviral vector pAIP (see below), encoding either GM-CSF or IL-4, and puromycin acetyltransferase. After two weeks of selection in 10 μg/ml puromycin transduced cells were incubated for two weeks in complete RPMI-1640 to allow secretion and accumulation of cytokines in the medium. The activity of the cytokines was tested by comparing the conditioned medium to commercially available GM-CSF, used at a final concentration of 50 ng/ml, and IL-4, used at a final concentration of 25 ng/ml, (both from R&D Systems). The phenotype and response to pattern recognition receptors and IFNβ of differentiated MDDC was tested using fluorescein isothiocyanate (FITC)-conjugated monoclonal antibodies against CD1a and CD86, phycoerythrin (PE)-conjugated monoclonal antibodies against CD14 and CD80, and allophycocyanin (APC)-conjugated monoclonal antibodies against CD209 (DC-SIGN) and CD83 (all from Miltenyi Biotec).

Opti-MEM® I Reduced Serum Media was purchased from Invitrogen and used to dilute DNA for transfection. Ultrapure, *E. coli* K12 LPS was obtained from Invivogen. Recombinant, human IFN-β was obtained from PBL InterferonSource. Deoxynucleosides were purchased from Sigma-Aldrich (2′deoxyguanosine monohydrate, cat# D0901; thymidine, cat# T1895; 2′deoxyadenosine monohydrate, cat# D8668; 2′deoxycytidine hydrochloride, cat# D0776). A 100 mM stock solution was prepared by dissolving each of the four nucleotides at 100 mM in RPMI 1640 by heating the medium at 80°C for 15 min. If not state otherwise exogenous nucleosides were added to the cells diluted in the appropriate medium 2 hrs before reporter vector addition. CUL4A inhibitor MLN4924 was purchased from Active Biochem and proteasome inhibitor MG132 was purchased from Sigma-Aldrich.

### Plasmid DNAs

Vpx from HIV-2 ROD and Vpx from SIV_MAC_251 Vpx was codon optimized through services provided by Sloning Biotechnologies GmbH (Puchheim, Germany) and Microsynth AG (Balgach, Switzerland) and the glutamine to alanine mutation at position 76 was introduced as described [41]. SIV Vpr from De Brazza’s monkey (SIV_DEB_ CM5 Vpr (AY523866)), mustached monkey (SIV_MUS1_ CM1239 (EF070330)) and SIV Vpx from red-capped mangabeys (SIV_RCM_ Vpx (AAM34564.1)) were codon optimized by Genecust and cloned into empty pcDNA3.1(−) or a version expressing a N-terminal triple HA-tag using 5′ *XbaI* and 3′ *NotI* sites. DNA sequences are provided in Table 1.

**Table 1:** Codon optimized Vpx and Vpr sequences

**Accessory gene**	**Codon-optimized nucleic acid sequence**
**SIV**_**MAC-251 **_**Vpx**	atgagcgacccaagagaaagaatcccacctggaaatagcggcgaagaaactattggagaggctttcgagtggctgaatagaaccgtggaggagataaatagagaagctgtgaaccatctgcccagagagctgatcttccaagtgtggcaaaggagctgggagtattggcacgacgagcagggcatgtcccagagctatgtgaaatatagatatctgtgtctgatgcagaaggcactgttcatgcactgtaaaaagggctgtaggtgcctcggggaaggacatggggccggcggatggaggcccggcccacctcctccccctccccccggcctcgcatga
**HIV-2**_**ROD **_**Vpx**	atgacagatccacgagagaccgtacccccaggcaacagtggagaagaaaccattggcgaggcgttcgcatggctcaacaggacggtggaggccatcaacagagaagccgtaaatcacctgcccagggaacttatctttcaggtctggcagaggagctggcggtactggcacgacgagcagggcatgtctgagagctataccaaataccgctacctttgtatcatccagaaggccgtttacatgcacgtgagaaaaggatgtacatgcttgggaagaggtcacggccctggcggctggagacctggcccaccaccccctcccccacctgggctggtgtga
**SIV**_**RCM **_**Vpx**	atggctgagcgggcaccagaagtgccaactggcgccggcgaggccgagtttcagccctggctccgggacatgttggagaaagtcaacctggaggcccggttgcacttccaccccgaattcatctttaggctgtggagaacatgcgtcgagcactggcatgatgtgcaccagaggtccctggagtacgccgcctataggtacctcctcctgatgcagaaggccctgttcattcactgccagaccgggtgtagccaaagacatgggcccaatcctagggctgtgggagagcgcattacaatcctgcctgggatgtga
**SIV**_**MUS1CM1239 **_**Vpr**	atggagagggtgcccccatcacatcggcccccatggcactccagggtggtcccaactaccatgcagcaggcacagcaggctatgtgggacctgaacgaggaagccgagaagcacttcagcagagaggagctgcggggaatctggaacgatgtcaccgagctccccgccgatcccaactggaccgtggatcaggccgctattgcctgtgccattgattacattcggcggactcagacactcctgtttcggcactacagggaaggctgctatcaccggtacagcaacacaatccgcaggtaccctaacatcagacccttgcgcgggacacaagcccctcccagtaacagcatgccaaatgccgaccctacacctccccttagaccctctaggtacaggatggacgagtga
**SIV**_**DEBCM5 **_**Vpr**	atggagcgctatcctcctagtcatcccccacatttcacatccagaactgtcccaatgacccggctggcactgcagcaggccatgcaggacctgaacgaggaggccctgaagcacttcaccagggaagagttgtggggggtgtggaaccactgtgtcgatttgcccgcccagcccgattggacaggagagcaggcctgggccgctagcgtgatcgattacattaaaatcgtgcaaaggatgctctggctccaccttagggaggcttgctttcaccgggagagagaggccacacggcggtaccccaacattaggccactgaccggccggaatagggaggtgagagacggggaatga

The following virus plasmids and vectors were used in this study: pWPTs-GFP is an HIV-1-based transfer vector with EGFP expression under the control of the EF1α promoter used in Figures 3A and 3B and 4F [60]. psPAX2, an HIV-1 *gag-pol* expression plasmid, and pMD2-G, a vesicular stomatitis virus G protein expression plasmid, were generous gifts from Didier Trono (Ecole Polytechnique Fédérale de Lausanne, Lausanne, Switzerland). 8.9NdSB is a minimal HIV-1 packaging plasmid used in Figure 3A and 3B [61]. pNL4-3-GFP is pNL4-3 with an *env*-inactivating mutation and GFP coding sequence in place of *nef* and used if not stated otherwise [62]. SIV3+ is an SIVmac251 *gag-pol* expression plasmid that also encodes Vpx, Vpr, and Vif used in Figure 3A and 3C [63]. SIV3+ ΔVpx was generated by digestion with BstB1 and religation after blunting ends with DNA Polymerase I, Large (Klenow) Fragment (New England BioLabs); this introduces a nonsense codon in place of Vpx amino acid 25. SIV_MAC_239*env*^–^GFP was used in Figure 3F and is described elsewhere [64] as well as psGAE used in Figure 3A and 3C [65], an SIV_MAC_ transfer vector expressing GFP, in which the cytomegalovirus (CMV) promoter was replaced with the spleen focus-forming virus (SFFV) LTR. pAPM is an HIV-1 based knockdown vector in which a single transcript driven by the SFFV LTR contains a miR30 framework modified to target a gene of interest and the puromycin N-acetyltransferase gene [50]. pAIP is an HIV-1-based transfer vector expressing the protein of interest from the SFFV LTR followed by the encephalomyocarditis virus (EMCV) internal ribosome entry site (IRES) cassette and the puromycin resistance cassette [66].

### Reporter vector and virus like particle production

Lentiviral vectors were produced by transfecting 293FT cells using Lipofectamin according to the protocol from the manufacturer (Invitrogen). For three part vector systems, the following DNA ratio was used: 4 parts transfer vector: 3 parts *gag-pol* expression plasmid: 1 part VSV-G expression plasmid. For two part virus systems a 7:1 ratio was used (7 parts *env*^–^ virus: 1 part pMD2.G). Virus like particles (VLP) were produced at a ratio of 5 parts pSIV3+ ΔVpx: 2 parts of Vpx expressing plasmid, and 1 part VSV-G expression plasmid. Viruses, vectors and VLP stocks were normalized by single cycle infectivity assay on HEK-293 or HeLa cells and by measuring the reverse transcriptase activity in the viral supernatant by qRT-PCR [67]. All supernatants were filtered through a 0.45 μm filter. For infections, virus stock was diluted in the appropriate cell culture medium and added to the cells. Vpx-VLPs were produced in the same manner and where added if not stated otherwise 2 hrs before addition of the reporter vectors used.

### RNAi and transgene expression in MDDCs

Knock down vectors targeting SAMHD1 were cloned as described previously [50]. Briefly, three 97-mer oligonucleotides (Table 2) were synthesized and PAGE purified by Microsynth AG (Balgach, Switzerland). Target sequences were cloned into pAPM using *EcoRI* and *XhoI* sites. The sequences were tested in HEK cells and ts 2 was determined by western blot analysis to be the most efficient in knocking down SAMHD1 and used in further experiments. To obtain knockdown in MDDC, CD14^+^ cells, freshly isolated from PBMC as described above, were treated with SIV_MAC_-251 VLP for 2 h, and then transduced with either a control or experimental pAPM microRNA-based shRNA vectors [41,50,51]. The CD14^+^ cells were then allowed to differentiate into MDDC. After differentiation, the MDDC were selected with 10 μg/ml puromycin for 24 h and assayed for protein knock down by western blot. Transduction efficiency was tested and determined to be greater than 90% of transduced MDDC using pAGM, a vector expressing EGFP in place of the puromycin selection cassette. For transient siRNA transfection targeting SAMHD1 in MDDC, 5 × 10^5^ MDDC were plated in a 12-well plate in 600 μl culture medium. 20 nM of siSAMHD1 ts2 (5′-CAACCAGAGCUGCAGAUAA-3′) (kindly provided by Dr. Nadine Laguette, CNRS Montpellier) were complexed with 6 μl of HiPerFect Transfection Reagent (Qiagen), following the manufacturer’s instructions and added to the MDDC culture. A second round of transfection was performed 24 hours later. Protein knock down was assessed by western blot 24 hours after the second round of transfection [68]. To over express codon optimized SIV Vpr and Vpx in MDDC the genes were cloned 5′ *XbaI* to 3′ *NotI* into a lentiviral vector (pscALPS) expressing the gene of interest under the control of the SFFV LTR and puromcyine selection cassette under the control of the CypA promoter. Freshly isolated CD14^+^ monocytes were treated with 2.5 mM deoxynucleoside mix (1.25 mM deoxyguanosine) for 2 hr and then freshly produced lentiviral vector supernatant was added in a 1:2 ratio. The cells were then allowed to differentiate into MDDC for 4 days and selected with 1 μg/ml puromycine for 24 hours. At day 5 of differentiation the cells were harvested and the deoxynucleosides were washed away with PBS before the cells were reseeded into 24 well plates for downstream experiments.

**Table 2:** shRNA SAMHD1 knockdown target sequences

	**SAMHD1 shRNA target sequences**
**TS1**	5′TGCTGTTGACAGTGAGCGCGCTTCCTTTATGAGATAGTATTAGTGAAGCCACAGATGTAATACTATCTCATAAAGGAAGCTTGCCTACTGCCTCGGA-3′
**TS2**	5′TGCTGTTGACAGTGAGCGCGCTGATTCGAGTATATTGTAATAGTGAAGCCACAGATGTATTACAATATACTCGAATCAGCTTGCCTACTGCCTCGGA-3′
**TS3**	5′TGCTGTTGACAGTGAGCGCGCCATCATCTTGGAATCCAAATAGTGAAGCCACAGATGTATTTGGATTCCAAGATGATGGCATGCCTACTGCCTCGGA-3′

### Real-time PCR for late RT, 2-LTR circles and integrated provirus

Low molecular weight DNA was extracted from 1 to 2×10^6^ MDDC using the DNA Blood and Tissue Kit from Qiagen. Quantitative PCR for NL4-3GFP (two part vector) or pWPTs (three part vector) late RT (full length HIV-1 cDNA, LRT) and 2-LTR circles overlapping the junction were detected with SYBRgreen (Invitrogen) or TaqMan probes as described here [52] 6 hours and 24 hours post infection, respectively. Primers to detect pWPTs-GFP LRT product using SYBRgreen was pWPTS J1B fwd and J2 rev (Table 3). Primers to detect 2-LTR circles overlapping the perfect junction for both pNL4-3-GFP and pWPTs-GFP using SYBRgreen were Junct2 fwd coupled with J2 rev. LRT products from pNL4-3-GFP were detected with the TaqMan system using the primers J1 fwd and J2 rev with the Late RT probe (LRT-P). 2-LTR circles overlapping the junction from pNL4-3-GFP were detected using the TaqMan sytem (Table 4) using the primers MH535 (fwd) and MH536 (rev) [69]. The TaqMan probes overlapping with the junction was JunctPro [52]. Integrated provirus of pNL4-3-GFP was detected with the TaqMan system using the Alu PCR primers MH535 (fwd), SB704 (rev) and MH603 probe [69]. Mitochondrial DNA was used for normalization with the following primer/probe set: Mito fwd (MH533), Mito rev (MH534), Mito probe [69]. SYBRgreen 2-LTR circle and LRT PCR reaction contained 1×SYBR green mix (10 mM Tris pH 8.3, 10 mM KCl, 2.5 mM NH_4_SO_4_, 5 mM MgCl_2_, 0.1 mg/ml BSA, 0.2 mM dNTPs, 1× SYBRgreen (Milford)), 300 nM each primer, 6 μl of template low-molecular weight DNA (100 to 250 ng total), and 0.1 μl of Hot Start Taq Polymerase (Promega) in a volume of 20 μl. After initial incubation at 95°C for 2 min to activate the Hot Start Taq Polymerase, 40 cycles of amplification and acquisition were carried out at 95°C for 6 s, followed by 10 s at 55°C, 30 s at 72°C and 6 s at 80°C. TaqMan 2-LTR circle PCR and LRT reaction mix contained 1× TaqMan Universal Master Mix (Applied Biosystems), 50 nM each primer, 100 nM TaqMan probe and 6 μl of template low-molecular weight DNA (100 to 250 ng total) in a volume of 20 μl. After an initial incubation at 95°C for 10 min, 50 cycles of amplification were carried out at 95°C for 15 s followed by 1 min and 30 s at 60°C. Alu PCR reaction mix contained 1× TaqMan Universal Master Mix (Applied Biosystems), 50 nM primer forward and 900 nM reverse primer, 100 nM TaqMan probe and 6 μl of template low-molecular weight DNA (100 to 250 ng total) in a volume of 20 μl. After an initial incubation at 95°C for 10 min, 50 cycles of amplification were carried out at 95°C for 15 s followed by 1 min and 30 s at 60°C Real-Time PCR reactions were run on a CFX96™ thermal cycler (Biorad).

**Table 3:** Oligonucleotides used for pWPTs-GFP quantitative PCR (De Iaco 2012)

	**Primer name**	**Primer sequence**
**Late RT**	pWPT J1B fwd	5′-GCATACATTATACGAAGTTATGCTGC-3′
	pWPT J2 rev	5′-GCCGTGCGCGCTTCAGCAAGC-3′
**2-LTR**	Junct2 fwd	5′-CAGTGTGGAAAATCTCTAGCAGTAC-3′
	pWPT J2 rev	5′-GCCGTGCGCGCTTCAGCAAGC-3′
**Alu PCR**	pWPT J1B fwd	5′-GCATACATTATACGAAGTTATGCTGC-3′
	SB704 rev	5′-TGCTGGGATTACAGGCGTGAG-3′
	MH603 probe	5′(FAM)-ACACTACTTGAAGCACTCAAGGCAAGCTTT-(TAMRA)3′

**Table 4:** Oligonucleotides used for NL4.3 GFP E- quantitative PCR (De Iaco, 2012 and Butler, 2001)

	**Primer name**	**Primer sequence**
**Late RT**	J1 fwd	5′-ACAAGCTAGTACCAGTTGAGCCAGATAAG-3′
	J2 rev	5′- GCCGTGCGCGCTTCAGCAAGC-3′
	LRT-P	5′-(FAM)-CAGTGGCGCCCGAACAGGGA-(TAMRA)-3′
**2-LTR**	MH535	5′-AACTAGGGAACCCACTGCTTAAG-3′
	MH536	5′-TCCACAGATCAAGGATATCTTGTC-3′
	JunctPro	5′- (FAM)-CTCTAGCAGTACTGGAAGGGCTA-(TAMRA)-3′
**Alu PCR**	MH535	5′-AACTAGGGAACCCACTGCTTAAG-3′
	SB704 rev	5′-TGCTGGGATTACAGGCGTGAG-3′
	MH603 probe	5′-(FAM)-ACACTACTTGAAGCACTCAAGGCAAGCTTT-(TAMRA)-3′
**Mito. DNA**	MH533	5′-ACCCACTCCCTCTTAGCCAATATT-3′
	MH534	5′-GTAGGGCTAGGCCCACCG-3′
	Mito probe	5′-(TET)-CTAGTCTTTGCCGCCTGCGAAGCA-(TAMRA)-3′

### Western blot

For western blot analysis 2 × 10^6^ HEK cells and 0.5 × 10^6^ to 1 × 10^6^ MDDCs were lysed in 200 μl and 50 μl 1% Triton lysis buffer (1% Triton X-100 (v/v), 50 mM Tris–HCl, pH 7.4 and 150 mM NaCl, supplemented with complete mini EDTA-free protease inhibitor cocktail tablets (Roche Applied Science)), respectively. Cells were lysed for 10 min on ice and centrifuged for 10 min at 14000 × g. The supernantant was transferred to a new tube, mixed with 2× Laemmli sample buffer (62.5 mM Tris, pH 6.8, 2% SDS, 10% glycerin, 715 mM β-mercaptoethanol and 0.001% bromophenol blue), supplemented with 2 mM EDTA and boiled at 100°C for 5 min. 15 μl were loaded onto a 12% SDS-PAGE. After SDS-PAGE, proteins were transferred onto an Immuno-Blot polyvinylidene fluoride (PVDF) membrane for 90 min at 110 V constant. The following antibodies were used in this study: anti-β-actin (Cat# M4439) and anti-GAPDH (Cat#G8795) were from Sigma-Aldrich. Polyclonal rabbit antibodies against MX1 (Cat# 13750-1-AP) and SAMHD1 (Cat# 12586-1-AP9) were purchased from ProteinTech Group. The following reagents were obtained through the AIDS Research and Reference Reagent Program, Division of AIDS, NIAID, NIH: HIV-2 Vpx Monoclonal Antibody (6D2.6) from Dr. John C. Kappes [70] and the Monoclonal Antibody to HIV-1 p24 (AG3.0) from Dr. Jonathan Allan [71]. Secondary antibodies HRP-linked donkey anti-rabbit IgG or HRP-linked sheep anti-mouse IgG were purchased from GE Healthcare Life Sciences. ECL or ECL Plus™ Western Blotting Detection Reagents (GE Healthcare Life Sciences) was used to reveal HRP signal on a Fujifilm LAS-4000 camera. Images were analyzed using the Multi-Gauge software (Fujifilm) and GIMP (General public software license).

### Intracellular nucleotide concentration

To measure the intracellular concentration in MDDCs, 2 × 10^6^ cells were incubated with Vpx-VLPs or nucleosides for the indicated timepoints and then harvested. The cell pellet was washed twice with PBS and the cells were lysed by resuspending the pellet in ice cold 65% methanol (100 μl per 1 × 10^6^ cells) and vigorously vortexed for 2 min. The cell lysate was then incubate at 95°C for 3 min and spun for 3 min at 14′000 rpm. The 65% methanol solution was then transferred to a new tube and completely dried by speed vacuum centrifugation. The dNTP pellet was store at -70°C until nucleotide measurement assay was performed as described elsewhere [47].

## Abbreviations

VLP: Virus-like particle; MDDC: Monocyte-derived dendritic cell; MDM: Monocyte -derived macrophages; LPS: Lipopolysaccharide; PRR: Pattern recognition receptor; IFN: Interferon; dNTP: Deoxynucleotide triphosphate; RT: Reverse transcriptase; LTR: Long terminal repeat; GFP: Green fluorescent protein.

## Competing interests

The authors declare that they have no competing interests.

## Authors’ contributions

CR and JL conceived and designed the experiments. CR, DB, and BK performed the experiments. CR and JL analyzed the data and wrote the paper. All authors read and approved the final manuscript.

## Supplementary Material

Additional file 1: Figure S1 SAMHD1 degradation timecourse. MDDCs were stimulated or not with 100 ng/ml LPS for 24 hrs and then treated with Vpx-VLPs. Protein samples for western blot anaylsis were collected before Vpx-VLPs addtion (0 hrs) or 3 hrs, 9 hrs, 24 hrs and 48 hrs after addition. Western blot for SAMHD1 and Actin as loading control is shown **(A)**. SAMHD1 protein levels were quantified after normalizion to the loading control and the 0 hrs samples for either in the absence or presence of LPS was set as 100% **(B)**. Click here for file

Additional file 2: Figure S2 SAMHD1 degradation timecourse**.** MDDCs from two donors were stimulated or not with 100 ng/ml LPS for 24 hrs and then treated with Vpx-VLPs. Protein samples for western blot anaylsis were collected 24 hrs addition. Western blot for SAMHD1, MX1, GAPDH1 and Actin as loading control is shown **(A)**. SAMHD1 protein levels were quantified after normalizion to the loading control and the 0 hrs samples for either in the absence or presence of LPS was set as 100% **(B)**. Nucleotides were exctracted from one sample per contidion and the concenration of deoxyadenosine **(C)** and deoxythymidine triphosphates **(D)** was measured. Click here for file
