# Supplementary figures and images for: Vpx rescue of HIV-1 from the antiviral state in mature dendritic cells is independent of the intracellular deoxynucleotide concentration
Source: Retrovirology. 2014 Feb 1;11:12. doi: 10.1186/1742-4690-11-12 (PMC3923257; doi:10.1186/1742-4690-11-12)

## Slide 1
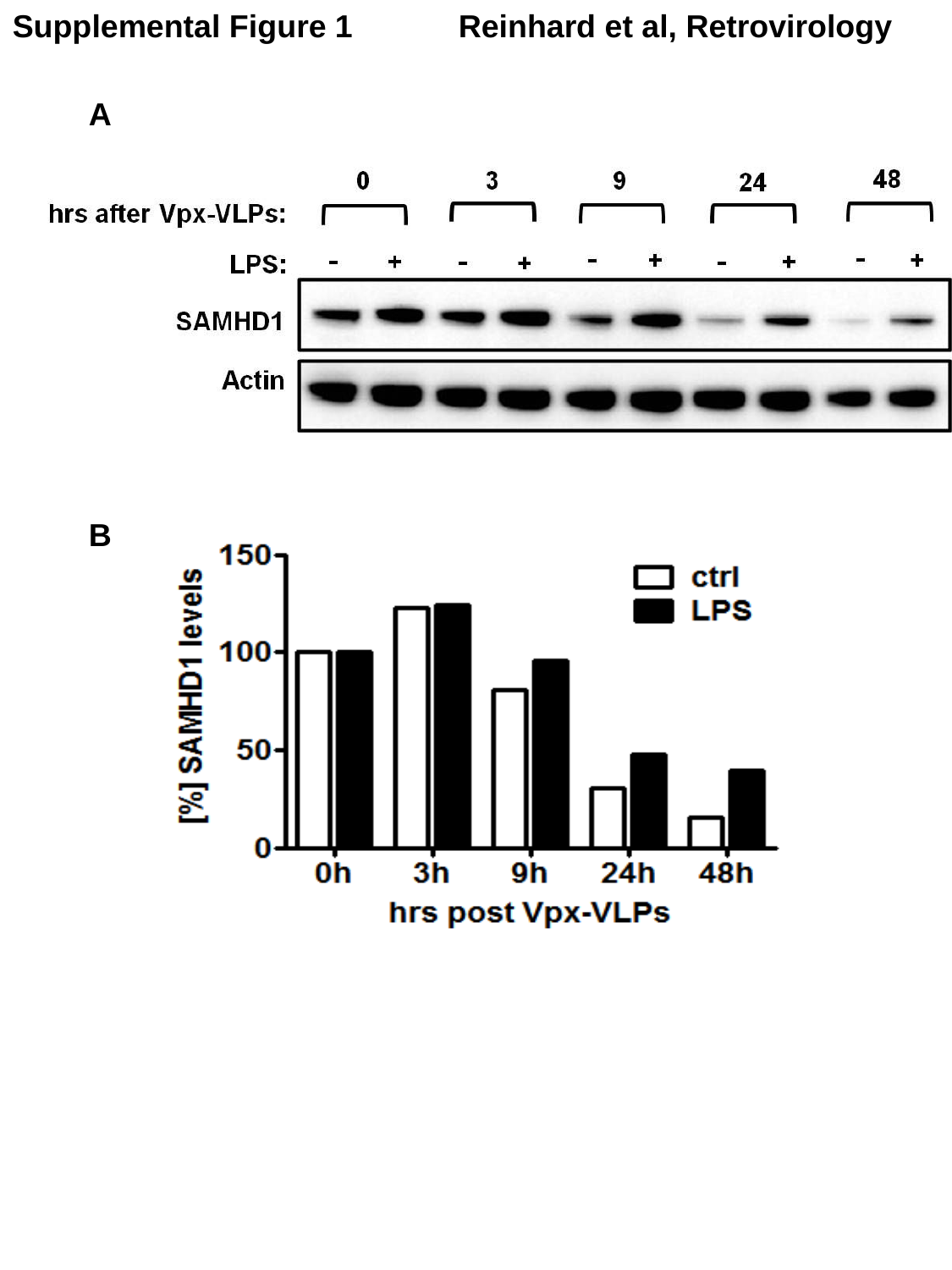

Supplemental Figure 1
Reinhard et al, Retrovirology
A
B

Supplement: Additional file 1: Figure S1 — SAMHD1 degradation timecourse. MDDCs were stimulated or not with 100 ng/ml LPS for 24 hrs and then treated with Vpx-VLPs. Protein samples for western blot anaylsis were collected before Vpx-VLPs addtion (0 hrs) or 3 hrs, 9 hrs, 24 hrs and 48 hrs after addition. Western blot for SAMHD1 and Actin as loading control is shown (A). SAMHD1 protein levels were quantified after normalizion to the loading control and the 0 hrs samples for either in the absence or presence of LPS was set as 100% (B). [file 1742-4690-11-12-S1.pptx]

## Slide 1
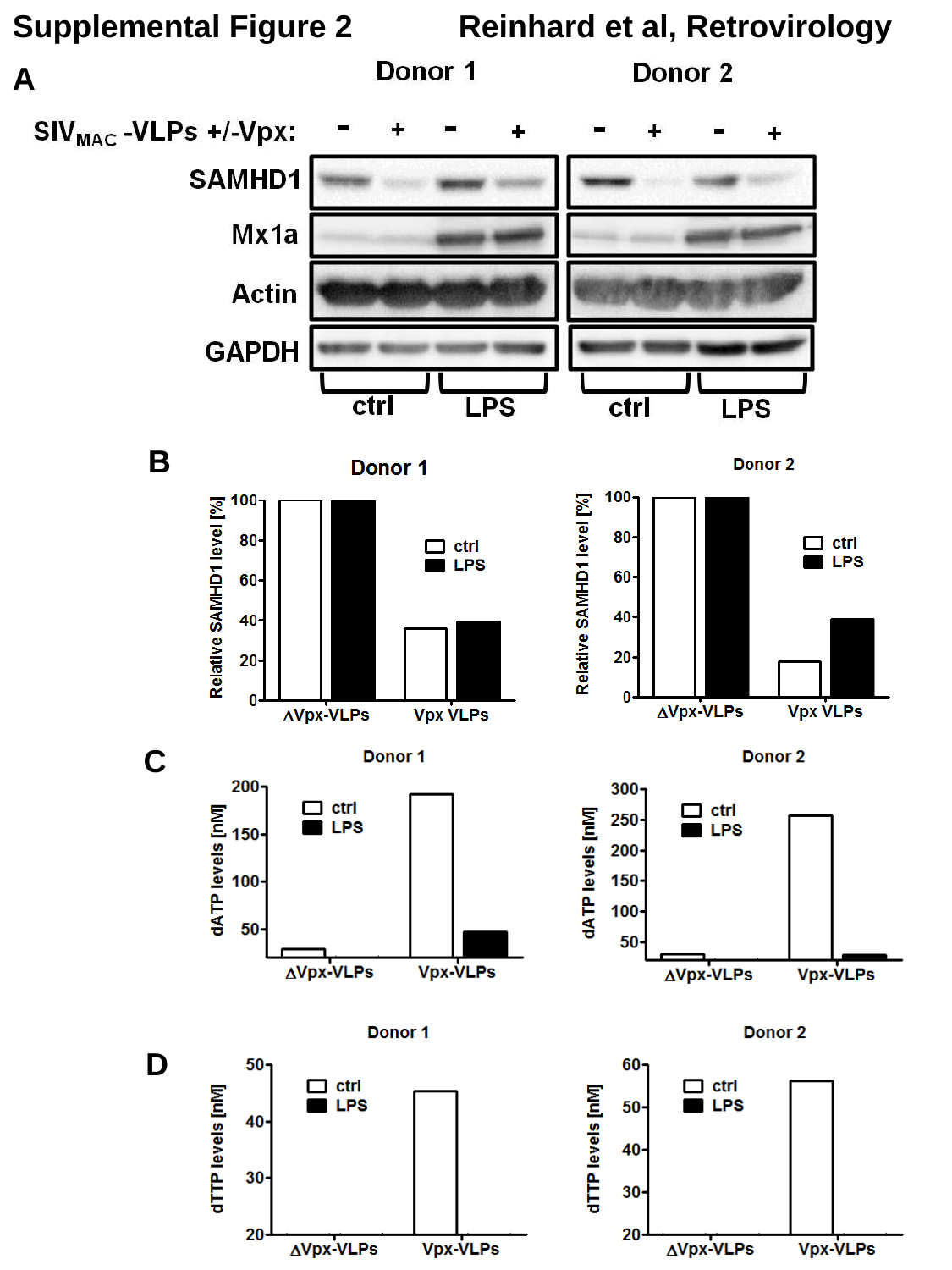

Supplemental Figure 2
Reinhard et al, Retrovirology
A
B
C
D

Supplement: Additional file 2: Figure S2 — SAMHD1 degradation timecourse. MDDCs from two donors were stimulated or not with 100 ng/ml LPS for 24 hrs and then treated with Vpx-VLPs. Protein samples for western blot anaylsis were collected 24 hrs addition. Western blot for SAMHD1, MX1, GAPDH1 and Actin as loading control is shown (A). SAMHD1 protein levels were quantified after normalizion to the loading control and the 0 hrs samples for either in the absence or presence of LPS was set as 100% (B). Nucleotides were exctracted from one sample per contidion and the concenration of deoxyadenosine (C) and deoxythymidine triphosphates (D) was measured. [file 1742-4690-11-12-S2.pptx]
